# Initial high-dose *β*-blockers toleration with reduced ejection fraction heart failure of hospitalized hypertensive patients

**DOI:** 10.3389/fcvm.2025.1672940

**Published:** 2025-12-15

**Authors:** Xinran Li, Xiangyu Yang, Yilin Zhou, Si Wang, Xiaoping Chen

**Affiliations:** 1 Department of Cardiology, West China Hospital, Sichuan University, Chengdu, Sichuan, China; 2 Department of Endocrinology and Metabolism, West China Hospital, Sichuan University, Chengdu, China

**Keywords:** heart failure with reduced ejection fraction, hypertension, initial high-dose beta-blockers, toleration, treatment strategy

## Abstract

**Background:**

*β*-blockers (BB) are the cornerstone of treatment for heart failure with reduced ejection fraction (HFrEF). However, due to potential adverse effects, guidelines recommend starting with a low dose and gradually titrating upwards. Hypertensive patients with HFrEF tend to have better drug responsiveness and prognosis, but it remains unclear whether they can tolerate initial high-dose BB. Therefore, this study aims to assess the tolerance of this population to initial high-dose BB therapy.

**Methods:**

A retrospective observational study included 307 hypertensive patients with HFrEF who initiated BB therapy and were admitted to the cardiology department at West China Hospital, Sichuan University. Patients’ demographic and clinical information was collected through the electronic medical record (EMR) system. Patients were categorized into a high-dose group if their initial BB dose exceeded 1/8 of the target dose, all other patients were assigned to the standard-dose group. Multivariate logistic forward regression analysis was performed to explore factors influencing the prescriptions of intial high-dose BB and adverse safety outcomes related to BB therapy during hospitalization, including bradycardia, hypotension, acute HF, wheezing requiring bronchodilator therapy, and BB dose reduction or cessation.

**Results:**

Seventy patients (22.8%) were initially prescribed high-dose BB. Logistic forward regression analysis revealed that only coronary heart disease was negatively associated with the prescriptions of initial high-dose BB, with an odds ratio of 0.435 (95% CI: 0.247–0.763, *P* = 0.004). Further logistic regression analysis demonstrated no independent association between the initial high-dose BB therapy and the occurrence of adverse safety outcomes, including bradycardia, hypotension, acute HF, wheezing requiring bronchodilator therapy, or BB dose reduction or discontinuation (all *p* < 0.05).

**Conclusion:**

Prescriptions of initial high-dose BB in hypertensive patients with HFrEF were not associated with an increased incidence of adverse safety outcomes. These findings indicate that initial high-dose BB therapy could be a viable strategy for this population.

## 1 Introduction

Heart failure (HF) represents the terminal stage of various cardiovascular diseases and is classified by ejection fraction (EF) into three categories: HF with reduced EF (HFrEF, EF < 40%), HF with mildly reduced EF (HFmrEF, EF 41%–49%), and HF with preserved EF (HFpEF, EF ≥ 50%) (1). Data from the China Hypertension Survey indicate an overall HF prevalence of 0.3% among 22,158 participants, with 40% having HFrEF, 23% with HFmrEF, and 36% with HFpEF (2). HF is associated with significant morbidity, mortality, and reduced quality of life. Among its various subtypes, HFrEF is linked to the poorest prognosis, HFrEF was associated with a nearly two-fold increased risk of 5 year mortality than HFpEF. Patients with HFrEF face a nearly two-fold higher risk of 5-year mortality compared to those with HFpEF (3).

The overactivation of the neuroendocrine system, including the sympathetic nervous system and the renin-angiotensin-aldosterone system, is a key pathophysiological mechanism in HFrEF (4). *β*-blockers (BB), essential neuroendocrine antagonists in the management of HFrEF, have robust evidence supporting their efficacy in improving patient outcomes (5). However, BB are often associated with adverse effects, such as bradycardia, hypotension, and bronchospasm, which may prevent patients from reaching target doses or lead to treatment discontinuation (6). As a result, clinical practice guidelines recommend a “start low, go slow” approach, initiating BB at low doses (no more than 1/8 of the target dose) and gradually titrating upwards (7). Despite this, some clinicians advocate for initial higher-dose BB during hospitalization to avoid delays or failures in uptitration after discharge, as general practitioners may be less familiar with their use in HF management (8, 9).

HF is a complex syndrome with multiple etiologies, leading to varied mechanisms of cardiac structural changes and prognosis (10–12). Accordingly, responses to pharmacological treatments may differ (13). Hypertension is a common cause of HF (1), and hypertension accounting for 39% of HF cases in men and 59% in women (14). Marco Bobbo et al. further observed that patients with hypertensive cardiomyopathy respond rapidly to optimal HF therapy and have a favorable cardiovascular prognosis (15). However, it remains unclear whether hypertensive patients with HFrEF can tolerate initial high-dose BB therapy.

Therefore, in this study, we aim to utilized inpatient data to investigate the association between initial high-dose BB therapy and the occurrence of adverse safety outcomes during hospitalization in hypertensive patients with HFrEF, providing a basis for further exploration of treatment strategies tailored to different HF etiologies.

## 2 Materials and methods

### 2.1 Study population

**Figure 1:**
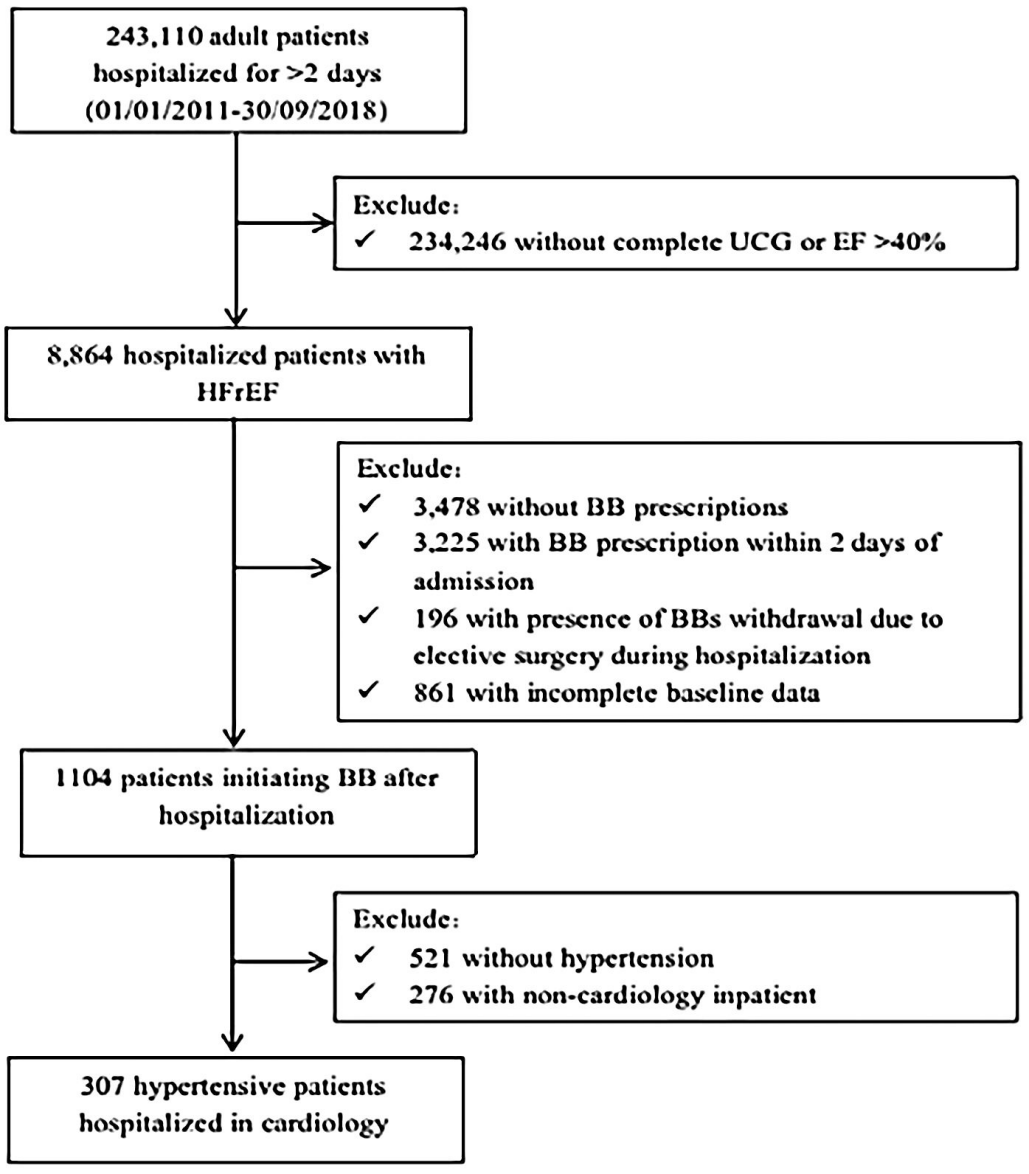
Flow chat. UCG, ultrasound cardiac imaging; EF, ejection fraction; HFrEF, heart failure with reduced ejection fraction; BB, beta-blockers.

We conducted a retrospective cohort study utilizing electronic medical records (EMRs) to identify hypertensive patients with HFrEF. The inclusion criteria were as follows: patients aged over 18 years who were hospitalized at West China Hospital of Sichuan University between January 1, 2011, and September 30, 2018; echocardiographic evidence of left ventricular ejection fraction (LVEF) <40%; a documented history of hypertension at discharge; admission to the cardiology department; and initiation of BB therapy during hospitalization, defined as starting treatment on or after the third day of admission (16). Exclusion criteria included a hospital stay of less than 2 days, incomplete laboratory or vital sign data, or patients who underwent any non-cardiac surgical procedures. A total of 307 hypertensive patients with HFrEF who initiated BB therapy were enrolled in the study (The inclusion and exclusion flowchart is shown in Figure 1). This study was approved by the Ethics Committee of West China Hospital, Sichuan University.

### 2.2 Data collection

We collected the following data from the inpatient EMR system: demographic information, including age, sex, smoking status, and alcohol consumption; vital signs, including systolic blood pressure (SBP), diastolic blood pressure (DBP), heart rate (HR) and respiratory rate; physical measurements, including height and weight; left ventricular ejection fraction (LVEF) from echocardiographic data; prescription records; laboratory test results; and discharge diagnoses based on the International Classification of Diseases, 10th Revision (ICD-10 codes). Body mass index (BMI) was calculated as weight divided by height squared (kg/m2). Baseline data were defined using prescription records from the first two calendar days following admission, the earliest recorded vital signs, and the initial laboratory test results. The laboratory test results included fasting blood glucose (FBG), total cholesterol (TC), triglycerides (TG), high-density lipoprotein cholesterol (HDL-C), low-density lipoprotein cholesterol (LDL-C), white blood cells (WBC), hemoglobin (Hb), N-terminal pro B-type natriuretic peptide (NT-proBNP), cardiac troponin T (cTnT), uric acid, and creatinine. Discharge diagnoses, including hypertension, diabetes mellitus (DM), coronary heart disease (CHD), arrhythmia, cardiomyopathy, rheumatic heart disease, and acute myocardial infarction (AMI), were identified using ICD-10 codes I10–I15, E10–E14, I20–I25, I44–I49, I42, I01–I09, and I21–I23, respectively. For patients with multiple hospitalizations, we analyzed data from their first admission.

### 2.3 Initiating dose of BB

The standardized initiating dose for each patient was determined by dividing the original initiating dose documented in the EMR by the target dose of BB. According to recent guidelines, patients were categorized into a high-dose group if their standardized initiating dose exceeded 1/8 of the target dose, while all other patients were assigned to the standard-dose group (7).

### 2.4 Adverse safety outcomes

According to the study by Zhou et al. (16), the adverse safety outcomes associated with BB therapy include bradycardia (defined as a HR below 60 beats per min after BB administration), hypotension (characterized by a SBP below 90 mmHg following BB treatment), acute HF (indicated by the administration of morphine or intravenous milrinone post-BB administration), wheezing requiring bronchoconstriction (defined as receiving intravenous methylprednisolone, aminophylline, or doxofylline after BB treatment), dose reduction events (described as a standardized BB dose lower than that of the previous day), and cessation events (defined as the discontinuation of BB therapy during hospitalization).

### 2.5 Statistical analysis

The study population's characteristics were summarized as frequencies and percentages for categorical variables, and as mean ± standard deviation (SD) for continuous variables. Differences between the two groups were analyzed using the Chi-square test for categorical variables, independent *t*-tests for normally distributed variables, and Mann–Whitney or Wilcoxon tests for non-normally distributed variables. Multivariate logistic forward regression analysis was used to explore the factors influencing the prescriptions of initial high-dose BB and adverse safety outcomes related to BB therapy during hospitalization in hypertensive patients with HFrEF. Univariate regression analyses were conducted for each independent variable and the dependent variable, and variables with *p*-values less than 0.15 and the initial high-dose BB therapy were included in the multivariate logistic regression model. A significant difference was set at a *p*-value <0.05. All statistical analyses were conducted using SPSS 23.0.

## 3 Results

### 3.1 Baseline characteristics in the initial standard-dose and high-dose BB therapy groups

A total of 307 hypertensive patients with HFrEF admitted to the cardiology department were included in the analyses. The baseline characteristics of the patients were presented in Table 1. Seventy patients (22.8%) were prescribed BB at doses higher than the guideline recommendation, of which 48 (68.6%) were male. Compared to the initial standard-dose BB therapy group, patients in the high-dose group were younger, exhibited lower cTnT levels, showed a reduced prevalence of CHD and AMI, and had an increased prevalence of cardiomyopathy and rheumatic heart disease. For adverse safety outcomes during hospitalization, the high-dose BB therapy group exhibited a significantly higher proportion of BB dose reduction or cessation events. However, no significant differences were observed between the two groups with respect to bradycardia, hypotension, new episodes of acute HF, or wheezing requiring bronchodilator therapy. Further comparison of baseline characteristics stratified by gender is presented in Supplement Table 1. Among female patients, there was no significant difference in the incidence of adverse safety outcomes between the initial high-dose group and the low-dose group. In contrast, among male patients, the incidence of dose reduction or discontinuation was higher in the high-dose group than in the low-dose group.

**Table 1:** Baseline characteristics in the initial standard-dose and high-dose BB therapy groups.

Variables	Standard- dose group	High-dose group	*p*-Value
*N* (%)	237 (77.2)	70 (22.8)	
Age, years	67.2 ± 12.1	63.9 ± 11.1	**0** **.** **045** *
Male, *n* (%)	159 (67.1)	48 (68.6)	0.816
Smoking, *n* (%)	130 (54.8)	38 (54.2)	0.933
Alcohol use, *n* (%)	87 (36.7)	28 (40)	0.617
BMI, kg/m^2^	23.4 ± 3.8	24.0 ± 3.4	0.364
HR, beats/min	90.0 ± 22.4	88.3 ± 25.0	0.583
SBP, mmHg	128 ± 23.3	132.4 ± 20.2	0.158
DBP, mmHg	78.8 ± 16.3	80.9 ± 18.2	0.364
FBG, umol/L	8.1 ± 3.9	8.7 ± 4.7	0.272
TG, mmol/L	1.43 ± 0.86	1.50 ± 0.91	0.55
TC, mmol/L	3.89 ± 1.08	3.77 ± 1.02	0.398
HDL-C, mmol/L	1.09 ± 0.35	1.12 ± 0.32	0.521
LDL-C, mmol/L	2.26 ± 0.89	2.11 ± 0.84	0.204
Uric Acid	428.5 ± 146.6	439.2 ± 144.2	0.59
Creatine	115.2 ± 74.2	109.2 ± 47.5	0.527
WBC	9.2 ± 5.3	8.3 ± 3.8	0.216
Hb, g/L	123.0 ± 20.9	131.6 ± 16.7	0.56
NT-proBNP, pg/mL	7,024.6 ± 6,399.0	6,474.4 ± 7,373.7	0.542
cTnT, ng/L	927.9 ± 1,709.00	459.9 ± 1,370.6	**0**.041*
LVEF (%)	31.2 ± 5.7	32.0 ± 5.0	0.255
Antihypertensive drugs			
ACEI, *n* (%)	126 (53.1)	51 (44.2)	0.570
ARB, *n* (%)	88 (37.1)	35 (50.0)	0.054
CCB, *n* (%)	72 (30.3)	25 (35.7)	0.399
Venous furosemide, *n* (%)	178 (75.1)	47 (67.1)	0.186
Oral furosemide, *n* (%)	80 (33.7)	25 (35.7)	0.761
Oral thiazide, *n* (%)	33 (13.9)	12 (17.1)	0.503
Aldosterone receptor antagonist, *n* (%)	202 (85.2)	54 (77.1)	0.110
Comorbidities			
Diabetes, *n* (%)	101 (42.6)	32 (45.7)	0.646
CHD, *n* (%)	174 (73.4)	37 (52.8)	**0.001** ^#^
AMI, *n* (%)	82 (34.5)	12 (17.1)	**0.005** ^#^
Cardiomyopathy, *n* (%)	23 (9.7)	12 (18.5)	**0.043** *
Rheumatic heart disease, *n* (%)	5 (2.1)	5 (7.1)	**0**.037*
Arrhythmia, *n* (%)	91 (38.3)	36 (51.4)	0.052
Adverse safety Outcome during Hospitalization			
Bradycardia, *n* (%)	62 (26.2)	24 (34.3)	0.183
Hypotension, *n* (%)	36 (15.2)	10 (14.3)	0.852
Acute HF, *n* (%)	26 (11.0)	10 (14.3)	0.449
Wheezing needing bronchoconstriction, *n* (%)	20 (8.4)	8 (11.4)	0.445
Dose reduction or cessation, *n* (%)	86 (36.3)	35 (50.0)	**0.039** *

n, number; BMI, body mass index; CHD, coronary heart disease; AMI, acute myocardial infarction; HR, heart rate; SBP, systolic blood pressure; DBP, diastolic blood pressure; FBG, fasting blood glucose; TC, total cholesterol; TG, triglycerides; HDL-C, high-density lipoprotein cholesterol; LDL-C, low-density lipoprotein cholesterol; WBC, white blood cells; Hb, hemoglobin; NT-proBNP, N terminal pro B type natriuretic peptide; cTnT, cardiac troponin T; LVEF, Left Ventricular Ejection Fractions; ACEI, angiotensin-converting enzyme inhibitors; ARB, angiotensin II receptor blockers; CCB, calcium channel blockers; BB, *β*-blockers; HF, heart failure.

A *P*-value < 0.05 was considered as statistically significant.

* *P* < 0.05.

# *P* < 0.001.

### 3.2 Logistic regression analysis for influencing factors of initial high-dose BB prescription

Logistic forward regression analysis was used to identify the factors influencing the initial high-dose BB prescription. Variables with *p*-values less than 0.15 from the univariate regression analysis, including age, CHD, AMI, cardiomyopathy, rheumatic heart disease, arrhythmia, SBP, cTnT, ARB, and aldosterone receptor antagonist, were integrated into the analysis. The results revealed that only CHD was negatively associated with initial high-dose BB prescription, with an OR value of 0.435 (95% CI: 0.247–0.763, *P* = 0.004). The results are presented in Table 2.

**Table 2:** Logistic regression analysis for influencing factors of initial high-dose BB prescription.

Independent Variable	Model 1	*p*-Value	Model 2	*p*-Value
Age	0.978 (0.957–1.000)	0.047	Not include	Not include
CHD	0.406 (0.234–0.704)	0.001	0.435 (0.247–0.763)	0.004*
AMI	0.391 (0.199–0.769)	0.007	Not include	Not include
Cardiomyopathy	2.122 (1.012–4.448)	0.046	Not include	Not include
Rheumatic heart disease	3.569 (1.003–12.706)	0.05	Not include	Not include
Arrhythmia	1.699 (0.993–2.906)	0.053	Not include	Not include
SBP	1.009 (0.997–1.021)	0.158	Not include	Not include
cTnT	1.000 (1.000–1.000)	0.051	Not include	Not include
ARB	0.055 (0.989–2.898)	0.055	Not include	Not include
Aldosterone Receptor Antagonist	0.113 (0.301–1.135)	0.113	Not include	Not include

CHD, coronary heart disease; AMI, acute myocardial infarction; SBP, systolic blood pressure; cTnT, cardiac troponin T; ARB, angiotensin II receptor blockers; BB, *β*-blockers.

Model 1: The variables with a *p*-value less than 0.15 in the logistic univariate regression analysis was presented in Model 1.

Model 2: Logistic forward regression analysis adjusting for variables with *p*-values less than 0.15 from univariate analysis, including age, CHD, AMI, cardiomyopathy, rheumatic heart disease, arrhythmia, SBP, cTnT, ARB, aldosterone receptor antagonist.

A *P*-value <0.05 was considered as statistically significant.

* *P* < 0.05.

### 3.3 Logistic regression analysis for influencing factors of adverse safety outcomes during hospitalization

Logistic forward regression analysis was employed to identify the factors influencing adverse safety outcomes related to BB during hospitalization, including bradycardia, hypotension, acute HF, wheezing requiring bronchoconstriction, and BB dose reduction or cessation events. The initial high-dose BB therapy and variables with *p*-values less than 0.15 from the univariate regression analysis were integrated into the multivariate regression analysis. The results revealed the following associations: bradycardia events were positively correlated with arrhythmia (OR = 2.057, 95% CI: 1.196–3.537, *P* = 0.009), oral furosemide (OR = 1.929, 95% CI: 1.103–3.372, *P* = 0.021), and intravenous furosemide (OR = 3.817, 95% CI: 1.827–7.971, *P* < 0.001), while negatively correlated with WBC (OR = 0.911, 95% CI: 0.852–0.974, *P* = 0.006), as shown in Table 3; hypotension events were positively associated with AMI (OR = 2.563, 95% CI: 1.272–5.161, *P* = 0.008) and intravenous furosemide (OR = 3.044, 95% CI: 1.025–9.042, *P* = 0.045), while negatively correlated with SBP (OR = 0.977, 95% CI: 0.961–0.994, *P* = 0.008), as presented in the Table 4; acute HF events were positively associated with CHD (OR = 4.982, 95% CI: 1.645–15.082, *P* = 0.004), CCB (OR = 3.346, 95% CI: 1.556–7.196, *P* = 0.002), and intravenous furosemide (OR = 15.706, 95% CI: 2.080–118.613, *P* = 0.008), as presented in the Table 5; wheezing events were positively associated with CCB (OR = 2.490, 95% CI: 1.119–5.540, *P* = 0.025) and venous furosemide (OR = 5.739, 95% CI: 1.328–24.998, *P* = 0.020), as presented in the Table 6; BB Dose reduction or cessation events were positively correlated only with arrhythmia (OR = 2.321, 95% CI: 1.451–3.713, *P* < 0.001), as presented in the Table 7. It was worth noting that we have not found an independent correlation between the initial high-dose BB therapy and the occurrence of adverse safety outcomes.

**Table 3:** Logistic regression analysis for influencing factors of bradycardia events during hospitalization.

Independent Variable	Model 1	*p*-Value	Model 2	*p*-Value
Initial high-dose of BB	1.473 (0.831–2.610)	0.185	Not include	Not include
Male	0.607 (0.362–1.020)	0.059	Not include	Not include
Arrhythmia	2.124 (1.281–3.521)	0.003	2.057 (1.196–3.537)	0.009*
Rheumatic heart disease	4.069 (1.119–14.794)	0.033	Not include	Not include
FBG	0.924 (0.858–0.996)	0.038	Not include	Not include
LDL-C	0.757 (0.553–1.037)	0.083	Not include	Not include
Uric Acid	1.001 (1.000–1.003)	0.117	Not include	Not include
WBC	0.942 (0.885–1.002)	0.056	0.911 (0.852–0.974)	0.006*
ARB	1.762 (1.065–2.917)	0.028	Not include	Not include
Oral furosemide	1.820 (1.089–3.040)	0.022	1.929 (1.103–3.372)	0.021*
Venous furosemide	2.859 (1.459–5.601)	0.002	3.817 (1.827–7.971)	<0.001^#^
Oral thiazide	1.902 (0.986–3.670)	0.055	Not include	Not include
Aldosterone receptor antagonist	1.731 (0.825–3.634)	0.147	Not include	Not include

FBG, fasting blood glucose; LDL-C, low-density lipoprotein cholesterol; WBC, white blood cells; ARB, angiotensin II receptor blockers; BB, *β*-blockers.

Model 1: The variables with a *p*-value less than 0.15 in the logistic univariate regression analysis was presented in Model 1.

Model 2: Logistic forward regression analysis adjusting for Initial high dose of beta-blockers and variables with *p*-values less than 0.15 from univariate analysis, including male, arrhythmia, rheumatic heart disease, FBG, LDL-C, uric acid, WBC, ARB, oral furosemide, venous furosemide, oral thiazide, aldosterone receptor antagonist.

A *P*-value < 0.05 was considered as statistically significant

* *P* < 0.05.

# *P* < 0.001.

**Table 4:** Logistic regression analysis for influencing factors of hypotension events during hospitalization.

Independent Variable	Model 1	*p*-Value	Model 2	*p*-Value
Initial high-dose of BB	0.931 (0.436–1.985)	0.852	Not include	Not include
Alcohol	1.656 (0.881–3.114)	0.118	Not include	Not include
CHD	4.389 (1.676–11.495)	0.003	Not include	Not include
AMI	3.690 (1.936–7.034)	<0.001	2.563 (1.272–5.161)	0.008*
SBP	0.979 (0.964–0.993)	0.004	0.977 (0.961–0.994)	0.007*
cTnT	1.000 (1.000–1.000)	0.009	Not include	Not include
Venous furosemide	3.432 (1.306–9.014)	0.012	3.044 (1.025–9.042)	0.045*

CHD, coronary heart disease; AMI, acute myocardial infarction; SBP, systolic blood pressure; cTnT, cardiac troponin T; BB, *β*-blockers.

Model 1: The variables with a *p*-value less than 0.15 in the logistic univariate regression analysis was presented in Model 1.

Model 2: Logistic forward regression analysis adjusting for Initial high dose of beta-blockers and variables with *p*-values less than 0.15 from univariate analysis, including alcohol, CHD, AMI, SBP, cTnT.

A *P*-value < 0.05 was considered as statistically significant.

* *P* < 0.05.

**Table 5:** Logistic regression analysis for influencing factors of acute HF events during hospitalization.

Independent Variable	Model 1	*p*-Value	Model 2	*p*-Value
Initial high-dose of BB	1.353 (0.618–2.961)	0.45	Not include	Not include
Male	0.434 (0.215–0.876)	0.02	Not include	Not include
Alcohol	1.794 (0.892–3.608)	0.101	Not include	Not include
Diabetes Mellitus	2.590 (1.258–5.331)	0.01	Not include	Not include
CHD	4.112 (1.411–11.98)	0.01	4.982 (1.645–15.082)	0.004*
Cardiomyopathy	0.193 (0.026–1.451)	0.11	Not include	Not include
AMI	2.566 (1.268–5.193)	0.009	Not include	Not include
HR	1.013 (0.999–1.027)	0.071	Not include	Not include
DBP	1.017 (0.997–1.038)	0.089	Not include	Not include
FBG	1.092 (1.018–1.171)	0.014	Not include	Not include
WBC	1.054 (0.994–1.118)	0.081	Not include	Not include
NT-proBNP	1.000 (1.000–1.000)	0.011	Not include	Not include
CCB	2.765 (1.366–5.598)	0.005	3.346 (1.556–7.196)	0.002*
Venous furosemide	14.921 (2.01–110.773)	0.008	15.706 (2.080–118.613)	0.008*

CHD, coronary heart disease; AMI, acute myocardial infarction; HR, heart rate; DBP, diastolic blood pressure; FBG, fasting blood glucose; WBC, white blood cells; NT-proBNP, N terminal pro B type natriuretic peptide; CCB, calcium channel blockers; BB, *β*-blockers; HF, heart failure.

Model 1: The variables with a *p*-value less than 0.15 in the logistic univariate regression analysis was presented in Model 1.

Model 2: Logistic forward regression analysis adjusting for Initial high dose of beta-blockers and variables with *p*-values less than 0.15 from univariate analysis, including male, alcohol, diabetes mellitus, CHD, cardiomyopathy, AMI, HR, DBP, FBG, WBC, NT-proBNP, CCB, venous furosemide.

A *P*-value <0.05 was considered as statistically significant.

* *P* < 0.05.

**Table 6:** Logistic regression analysis for influencing factors of wheezing events during hospitalization.

Independent Variable	Model 1	*p*-Value	Model 2	*p*-Value
Initial high-dose of BB	1.400 (0.588–3.332)	0.447	Not include	Not include
Alcohol	2.065 (0.945–4.514)	0.069	Not include	Not include
HR	1.013 (0.998–1.029)	0.084	Not include	Not include
Uric Acid	0.997 (0.994–1.000)	0.085	Not include	Not include
Creatine	1.004 (1.000–1.008)	0.006	Not include	Not include
WBC	1.069 (1.005–1.137)	0.034	Not include	Not include
CCB	2.361 (1.078–5.172)	0.032	2.490 (1.119–5.540)	0.025*
Venous furosemide	5.226 (1.212–22.536)	0.027	5.739 (1.318–24.998)	0.02*

HR, heart rates; WBC, white blood cells; CCB, calcium channel blockers; BB, *β*-blockers.

Model 1: The variables with a *p*-value less than 0.15 in the logistic univariate regression analysis was presented in Model 1.

Model 2: Logistic forward regression analysis adjusting for Initial high dose of beta-blockers and variables with *p*-values less than 0.15 from univariate analysis, including alcohol, HR, uric acid, creatine, WBC, CCB, venous furosemide.

A *P*-value <0.05 was considered as statistically significant.

* *P* < 0.05.

**Table 7:** Logistic regression analysis for influencing factors of BB dose reduction or cessation during hospitalization.

Independent Variable	Model 1	*p*-Value	Model 2	*p*-Value
Initial high-dose of BB	1.756 (1.025–3.007)	0.04	Not include	Not include
HR	1.010 (1.000–1.020)	0.048	Not include	Not include
Creatine	1.003 (0.999–1.006)	0.129	Not include	Not include
NT-proBNP	1.000 (1.000–1.000)	0.07	Not include	Not include
Arrhythmia	2.321 (1.451–3.713)	<0.001	2.321 (1.451–3.713)	<0.001^#^
Aldosterone receptor antagonist	1.898 (0.978–3.684)	0.058	Not include	Not include

HR, heart rate; NT-proBNP, N terminal pro B type natriuretic peptide; BB, *β*-blockers.

Model 1: The variables with a *p*-value less than 0.15 in the logistic univariate regression analysis was presented in Model 1.

Model 2: Logistic forward regression analysis adjusting for Initial high dose of beta-blockers and variables with *p*-values less than 0.15 from univariate analysis, including HR, Creatine, Arrhythmia, NT-proBNP, Aldosterone receptor antagonist.

A *P*-value < 0.05 was considered as statistically significant.

# *P* < 0.001.

## 4 Disscusion

This study found that among hypertensive patients with HFrEF, 22.8% were initially prescribed high-dose BB. Patients with CAD were less likely to be prescribed a high-dose BB. After adjusting for confounding variables, initial high-dose BB therapy was not associated with an increased incidence of adverse safety outcomes, including bradycardia, hypotension, acute HF, bronchoconstriction requiring bronchodilator therap, or BB dose reduction or cessation events.

Contrary to our findings, Zhou et al. reported that among 1,104 HFrEF patients who initiated BB therapy during hospitalization, 27.5% received high-dose BB treatment, which was associated with a higher risk of dose reduction or withdrawal (16). The discrepancies between these findings may be attributed to the varying etiologies of HF. HF is a heterogeneous condition, representing the final stage of numerous cardiac diseases, with hypertension being the most prevalent cause (1). Persistent hypertension leads to left ventricular hypertrophy and diastolic dysfunction through direct hemodynamic overload and indirect neuroendocrine mechanisms (14, 17). Over time, this pathological state, further aggravated by coronary microvascular dysfunction, myocardial fibrosis, and comorbidities such as diabetes and obesity, progresses to dilated cardiomyopathy with HFrEF (18). Compared to ischemic and dilated cardiomyopathy, hypertensive HF is associated with a better prognosis and a more favorable response to pharmacotherapy (15). Additionally, previous research has indicated that lower pre-treatment BP is associated with an increased risk of cardiovascular mortality (19, 20), but it did not perform subgroup analyses based on the etiology of heart failure. The improvement in clinical symptoms following HF treatment is often accompanied by an elevation in BP (21). These findings suggest that in patients with HFrEF, higher baseline BP may reflect better compensatory capacity of myocardial contractility (15). This may partially account for our observation that initial high-doses BB did not elevate the risk of adverse safety outcomes among hypertensive patients with HFrEF.

The REBOOT trial recently suggested that BB use may be harmful in women with MI and HFpEF, particularly at higher doses—a finding not observed in men (22). Unlike REBOOT, our study, while noting more frequent dose reduction or discontinuation in high-dose males, found no independent link between sex and adverse outcomes in multivariate analysis. This difference may arise from distinct patient cohorts: REBOOT reported a significant interaction between sex and BB use in patients with HFpEF, but not in those with HFmrEF, whereas we only included HFrEF, a group with marked neurohormonal activation. Moreover, our analysis was limited to the initial in-hospital BB dose without follow-up data. Further research is needed to clarify sex-based responses to BB dosing across heart failure subtypes.

Additionally, studies indicate that BB therapy can provide mortality and morbidity benefits within 2–3 weeks of initiation (23). Furthermore, in real-world clinical practice, community general practitioners may lack familiarity with the use of BB in HF management, potentially leading to delays or failures in the uptitration of therapy following hospital discharge. Therefore, some clinicians advocate for initial high-doses BB during hospitalization (8, 9). Overall, we hypothesize that initial high-dose BB therapy in hypertensive patients with HFrEF could improve outcomes, though further research is needed to confirm this.

There are several limitations to our study. First, as a retrospective analysis, medication data were collected from the inpatient EMR system, which may miss unrecorded self-administered medications, especially in non-cardiology wards. To reduce this bias, we limited the study to cardiology department patients. Additionally, the study focused solely on hospitalization data without follow-up information, which may not fully capture the compliance and effectiveness of long-term BB use. However, given the challenges of obtaining comprehensive data from real-world outpatient settings and the importance of initiating BB during hospitalization for long-term adherence, our analysis was limited to inpatient BB use. Lastly, the etiology of HF in the included patients may include ischemic or other causes, rather than being solely attributable to hypertension. Further research is needed to validate these findings in patients with HFrEF where hypertension is the sole underlying cause.

## 5 Conclusion

In conclusin, the initial high-dose BB therapy in hypertensive patients with HFrEF does not increase the risk of adverse safety outcomes, including bradycardia, hypotension, acute HF, bronchoconstriction requiring bronchodilator therapy, or BB dose reduction or cessation events. These findings suggest that a initial high dose BB therapy may be a feasible strategy in this population. Further research is warranted to explore the long-term benefits of initiating high-dose BB therapy.

## Data Availability

The datasets presented in this study can be found in online repositories. The names of the repository/repositories and accession number(s) can be found in the article/Supplementary Material.
