# Supplementary material for: Initial high-dose β-blockers toleration with reduced ejection fraction heart failure of hospitalized hypertensive patients
Source: Front Cardiovasc Med. 2025 Dec 15;12:1672940. doi: 10.3389/fcvm.2025.1672940 (PMC12745278; doi:10.3389/fcvm.2025.1672940)
Supplement: Supplementary file 1 [file Table1.docx]

| Supplement Table 1. Baseline characteristics in the initial standard-dose and high-dose BB therapy groups stratified by sex | | | | | | |
| --- | --- | --- | --- | --- | --- | --- |
|  | female | |  | male | |  |
| Variables | standard-dose group (n=78) | High-dose group (n=22) | P-value | standard-dose group (n=159) | High-dose group (n=48) | P-value |
| Age, years | 65.8±13.9 | 63.2±10.1 | 0.409 | 67.8±11.1 | 64.2±11.7 | 0.054 |
| Male, n (%) | 159 (67.1) | 48 (68.6) | 0.816 | 159 (67.1) | 48 (68.6) | 0.816 |
| Smoking, n (%) | 43 (55.1) | 13 (59.1) | 0.741 | 87 (55.1) | 25 (52.1) | 0.717 |
| Alcohol use, n (%) | 30 (38.5) | 13 (59.1) | 0.084 | 57 (35.8) | 15 (31.3) | 0.558 |
| BMI, kg/m^2^ | 23.4±4.26 | 24.8±3.4 | 0.327 | 23.3±3.6 | 23.7±3.4 | 0.712 |
| HR, beats/minute | 90.8±22.1 | 87.0±23.9 | 0.487 | 89.6±22.7 | 88.9±25.6 | 0.847 |
| SBP, mmHg | 125.7±20.5 | 132.2±19.1 | 0.187 | 129.1±24.5 | 132.4±20.9 | 0.398 |
| DBP, mmHg | 78.8±16.3 | 80.9±18.2 | 0.439 | 78.3±15.8 | 82.9±17.9 | 0.09 |
| FBG, umol/L | 8.7±4.4 | 7.2±3.2 | 0.15 | 7.8±3.6 | 9.4±5.1 | 0.018* |
| TG, mmol/L | 1.4±0.6 | 1.5±0.8 | 0.378 | 1.4±0.9 | 1.5±0.9 | 0.825 |
| TC, mmol/L | 3.8±0.9 | 3.9±0.9 | 0.656 | 3.9±1.2 | 3.7±1.1 | 0.223 |
| HDL-C, mmol/L | 1.1±0.3 | 1.1±0.4 | 0.647 | 1.1±0.4 | 1.1±0.3 | 0.643 |
| LDL-C, mmol/L | 2.2±0.7 | 2.2±0.8 | 0.909 | 2.3±1.0 | 2.1±0.9 | 0.168 |
| Uric Acid | 436.2±149.8 | 457.5±158.9 | 0.562 | 424.7±145.3 | 430.7±137.9 | 0.796 |
| Creatine | 114.3±54.8 | 116.1±59.5 | 0.892 | 115.6±82.2 | 106.1±41.1 | 0.439 |
| WBC | 8.9±4.2 | 8.9±3.8 | 0.913 | 9.4±5.8 | 8.2±3.9 | 0.181 |
| Hb, g/L | 131.4±23.8 | 130.8±17.3 | 0.916 | 129.2±19.3 | 131.9±16.5 | 0.394 |
| NT-proBNP, pg/mL | 7309.0±6871.4 | 7700.9±8646.2 | 0.824 | 6885.0±6171.8 | 5912.2±6737.4 | 0.35 |
| cTnT, ng/L | 871.5±1609.3 | 736.1±1719.3 | 0.734 | 955.7±1760.7 | 324.9±1162.0 | 0.025* |
| LVEF (%) | 30.4±5.8 | 33.0±4.5 | 0.051 | 31.6±5.6 | 31.6±5.1 | 0.992 |
| Antihypertensive drugs |  |  |  |  |  |  |
| ACEI, n (%) | 37 (47.4) | 9 (40.9) | 0.587 | 89 (56.0) | 22 (45.8) | 0.217 |
| ARB, n (%) | 36 (46.2) | 11 (50.0) | 0.75 | 52 (32.7) | 24 (50.0) | 0.029* |
| CCB, n (%) | 23 (29.5) | 9 (40.9) | 0.31 | 49 (30.8) | 16 (33.3) | 0.742 |
| Venous furosemide, n (%) | 64 (82.1) | 15 (68.2) | 0.158 | 114 (71.7) | 32 (66.7) | 0.503 |
| Oral furosemide, n (%) | 32 (41.0) | 5 (22.7) | 0.116 | 48 (30.2) | 20 (40.7) | 0.138 |
| Oral thiazide, n (%) | 13 (16.7) | 5 (22.7) | 0.513 | 20 (12.6) | 7 (14.6) | 0.718 |
| Aldosterone receptor antagonist, n (%) | 71 (91.0) | 15 (68.2) | 0.006* | 131 (82.4) | 39 (81.3) | 0.857 |
| Comorbidities |  |  |  |  |  |  |
| Diabetes, n (%) | 38 (48.7) | 8 (36.4) | 0.304 | 63 (39.6) | 24 (50.0) | 0.202 |
| CHD, n (%) | 61 (78.2) | 11 (50.0) | 0.009* | 113 (71.1) | 26 (54.2) | 0.029 * |
| AMI, n (%) | 27 (34.6) | 6 (27.3) | 0.518 | 55 (34.6) | 6 (12.5) | 0.003* |
| Cardiomyopathy, n (%) | 7 (9.0) | 4 (18.2) | 0.223 | 16 (10.1) | 9 (18.8) | 0.106 |
| Rheumatic heart disease, n (%) | 2 (2.6) | 3 (13.6) | 0.035* | 3 (1.9) | 2 (4.2) | 0.367 |
| Arrhythmia, n (%) | 31 (39.7) | 11 (50.0) | 0.389 | 60 (37.7) | 25 (52.1) | 0.077 |
| Adverse safety Outcome during Hospitalization |  |  |  |  |  |  |
| Bradycardia, n (%) | 26 (33.3) | 9 (40.9) | 0.511 | 36 (22.6) | 15 (31.3) | 0.225 |
| Hypotension, n (%) | 12 (15.4) | 5 (22.7) | 0.418 | 24 (15.1) | 5 (10.4) | 0.413 |
| Acute HF, n (%) | 13 (16.7) | 5 (22.7) | 0.513 | 13 (8.2) | 5 (10.4) | 0.629 |
| Wheezing needing bronchoconstriction, n (%) | 9 (11.5) | 2 (9.1) | 0.746 | 11 (6.9) | 6 (12.5) | 0.217 |
| Dose reduction or cessation, n (%) | 31 (39.7) | 10 (45.5) | 0.631 | 55 (34.6) | 25 (52.1) | 0.029 * |
| Abbreviations: n, number; BMI, body mass index; CHD, coronary heart disease; AMI, acute myocardial infarction; HR, heart rate; SBP, systolic blood pressure; DBP, diastolic blood pressure; FBG, fasting blood glucose; TC, total cholesterol; TG, triglycerides; HDL-C, high-density lipoprotein cholesterol; LDL-C, low-density lipoprotein cholesterol; WBC, white blood cells; Hb, hemoglobin; NT-proBNP, N terminal pro B type natriuretic peptide; cTnT, cardiac troponin T; LVEF, Left Ventricular Ejection Fractions; ACEI, angiotensin-converting enzyme inhibitors; ARB, angiotensin II receptor blockers; CCB, calcium channel blockers; β-blockers, BB; HF, heart failure; A P-value < 0.05 was considered as statistically significant; *P＜0.05；#P＜0.001； | | | | | | |
